# Black 3D-TiO_2_ Nanotube Arrays on Ti Meshes for Boosted Photoelectrochemical Water Splitting

**DOI:** 10.3390/nano12091447

**Published:** 2022-04-24

**Authors:** Ming Meng, Yamin Feng, Chunyang Li, Zhixing Gan, Honglei Yuan, Honghui Zhang

**Affiliations:** 1 School of Physics and Telecommunication Engineering, Zhoukou Normal University, Zhoukou 466001, China; yadan205@126.com (Y.F.); lichunyang98@163.com (C.L.); yuanhenu@163.com (H.Y.); zhanghonghui4714@163.com (H.Z.); 2 Key Laboratory of Optoelectronic Technology of Jiangsu Province, School of Physical Science and Technology, Nanjing Normal University, Nanjing 210023, China

**Keywords:** 3D-TiO_2_ nanotube arrays, electrochemical reduction, oxygen vacancies, photoelectrochemical water splitting

## Abstract

Black 3D-TiO_2_ nanotube arrays are successfully fabricated on the Ti meshes through a facile electrochemical reduction method. The optimized black 3D-TiO_2_ nanotubes arrays yield a maximal photocurrent density of 1.6 mA/cm^2^ at 0.22 V vs. Ag/AgCl with Faradic efficiency of 100%, which is about four times larger than that of the pristine 3D-TiO_2_ NTAs (0.4 mA/cm^2^). Such boosted PEC water splitting activity primarily originates from the introduction of the oxygen vacancies, which results in the bandgap shrinkage of the 3D-TiO_2_ NTAs, boosting the utilization efficiency of visible light including the incident, reflected and/or refracted visible light captured by the 3D configuration. Moreover, the oxygen vacancies (Ti^3+^) can work as electron donors, which leads to the enhanced electronic conductivity and upward shift of the Fermi energy level, and thereby facilitating the transfer and separation of the photogenerated charge carrier at the semiconductor-electrolyte interface. This work offers a new opportunity to promote the PEC water splitting activity of TiO_2_-based photoelectrodes.

## 1. Introduction

Photoelectrochemical (PEC) water splitting technology capable of directly converting and storing the abundant solar energy into energy-dense hydrogen fuel has emerged as a promising strategy to alleviate the worsening energy crisis and environmental issues [1,2,3,4,5,6,7,8,9]. To achieve the practical application of this technology, the fabrication of stable and efficient photoelectrodes are desperately needed [10,11,12,13,14,15,16,17]. Three-dimensional TiO_2_ nanotube arrays (3D-TiO_2_ NTAs) formed on Ti mesh have been recognized as a competitive candidate in the design and fabrication of a photoanode for PEC water splitting owing to its larger internal and external surface areas, efficient charge separation and transportation features, and optimal adhesion with Ti mesh [18,19,20,21,22,23,24,25,26,27,28]. More importantly, the 3D-TiO_2_ NTAs on Ti mesh exhibits significant improvement in the utilization efficiency of Ti source compared to the 2D-TiO_2_ nanotube arrays formed on Ti foil [18,20,28]. Besides, the radial nature of 3D-TiO_2_ NTAs endows it with capability of harvesting the incident, reflected and/or refracted ultraviolet and visible light from any direction surrounding the Ti wire, rendering a higher PEC water splitting activity to be achieved [18,20,29]. However, its PEC performances are still inhibited by the large bandgap (3.2 eV), which results in the photoexcited electron and hole not being produced by the visible light harvested by the 3D NTAs [30,31,32,33,34,35,36,37]. In addition, 3D-TiO_2_ NTAs also suffer from poor electrical conductivity, and the bulk and surface recombination of photogenerated charge carriers, both of which are detrimental to the PEC water splitting activity [37,38,39,40,41]. Consequently, seeking an efficient strategy to boost the utilization of visible light and the electrical conductivity is vitally crucial.

Lately, the introduction of the oxygen vacancies (O-vacancies) has been demonstrated as an effective tactic to steer the optical and electronic characteristics of the metal oxide [41,42,43,44]. As illustrated by many research groups, the introduction of the O-vacancies can enable the Fermi energy level to shift toward the conduction band, which leads to the shrinkage of the bandgap, thus promoting the utilization efficiency of the visible light [31,41,42,43,44]. In addition, the presence of O-vacancies can also increase the electrical conductivity due to the high donor density, which facilitates the separation and transport of photogenerated charge carriers [31,38,39,44]. Accordingly, it is anticipated that rational introduction of the O-vacancies in 3D-TiO_2_ NTAs may be a promising route to tackle the two abovementioned drawbacks. Unfortunately, the reported available strategies to produce O-vacancies generally involve the harsh experimental conditions or high-cost facilities, which are not suitable for the largescale practical application [31,38,39,44]. Hence, exploiting a simple and economical method to introduce O-vacancies into metal oxide still requires more endeavors.

Recently, an electrochemical reductive doping process has proved to be a simple and cost-effective route to introduce O-vacancies into the TiO_2_ NTAs [44,45,46,47,48]. Under an external electric field, the Ti^4+^ is reduced to Ti^3+^, which leads to the generation of O-vacancies. Three different reduction electrolytes have been utilized, including acidic (H_2_SO_4_), neutral (Na_2_SO_4_), and alkaline (KOH) aqueous solution [44,45,46,47,48,49]. It is found that the alkaline electrolytes are more favorable to the introduction of O-vacancies because of the occurrence of a gas-forming side reaction during reduction in acidic solution [47,49]. Nevertheless, the existing research mainly focused on electrochemical reduction in acidic and neutral aqueous solution. As such, electrochemical reduction in alkaline aqueous solution have not been comprehensively understood. For example, the fundamental questions are whether alkaline aqueous solution is general or just for KOH, which remains unclear so far.

Herein, black 3D-TiO_2_ NTAs with substantial O-vacancies were prepared via a simple electrochemical reduction in NaOH solution, where the 3D-TiO_2_ NTAs were reduced by cathodic polarization for 15 min. As expected, the optimally reduced 3D-TiO_2_ NTAs generated a photocurrent density of 1.6 mA/cm^2^ at 0.22 V vs. Ag/AgCl with Faradic efficiency of 100%, nearly four times higher than that of the pristine 3D-TiO_2_ NTAs. Such boosted PEC water splitting activity primarily originates from the introduction of the O-vacancies, which results in bandgap shrinkage of the 3D-TiO_2_ NTAs, boosting the utilization efficiency of visible light including the incident, reflected and/or refracted visible light captured by the 3D configuration. Moreover, the O-vacancies (Ti^3+^) can work as electron donors, which leads to enhanced electronic conductivity and upward shift of the Fermi level, thereby facilitating the transfer and separation of the photogenerated charge carrier at the semiconductor-electrolyte interface. This work offers a new opportunity to promote the PEC water splitting activity of TiO_2_-based photoelectrodes.

## 2. Materials and Methods

### 2.1. Preparation of the 3D-TiO_2_ NTAs

The 3D-TiO_2_ NTAs were fabricated by the electrochemical anodization of Ti meshes. Briefly, anodization was performed via a conventional two-electrode system, with clean Ti mesh (Alfa Aesar (China) Chemical Co. Ltd, Shanghang, China, 80-mesh) with size of 1.5 cm × 1 cm as the anode and Pt mesh as the cathode, respectively. The electrolytes solution was prepared by dissolving 0.3 wt% NH_4_F and 2 vol% DI H_2_O in ethylene glycol. The Ti mesh was anodized by 60 V for 1 h. After anodization, the as-prepared 3D-TiO_2_ NTAs were thoroughly rinsed with ethanol and DI H_2_O, respectively, and then were annealed in air at 400 °C for 2 h (denoted as pristine 3D-TiO_2_ NTAs).

### 2.2. Electrochemical Reduction of the 3D-TiO_2_ NTAs

The electrochemical reduction was conducted in a conventional three-electrode system. The as-prepared 3D-TiO_2_ NTAs, Ag/AgCl (3 mol L-1 KCl-filled) and Pt mesh were employed as the working, reference, and counter electrode, respectively. NaOH aqueous solution (1 M, pH = 13.6) was utilized as electrolyte. The electrochemical reduction bias of −1.2, −1.3 and −1.4 (vs. Ag/AgCl) were used, and the corresponding photoelectrodes are denoted as ECR-3D-TiO_2_ NTAs−x, where x = 1.2, 1.3 and 1.4 V. The time of the electrochemical reduction was 15 min.

### 2.3. Characterization

Morphologies, microstructures and crystal structures of the as-prepared samples were characterized by field-emission scanning electron microscopy (FE-SEM, S4800, Hitachi Ltd., Tokyo, Japan), field-emission transmission electron microscopy (FE-TEM, JEM-2100, JEOL Ltd., Tokyo, Japan) and X-ray powder diffractometry (XRD, Xpert, Philips, Amsterdam, The Netherlands). The diffuse reflectance spectra were measured by a VARIAN Cary5000 spectrophotometer (Varian, CA, USA). The X-ray photoelectron spectroscopy (XPS) data were collected by the PHI 5000 Versaprobe (Ulvac-Phi, Kanagawa, Japan).

### 2.4. Photoelectrochemical Measurements

The PEC tests were conducted in a three-electrode configuration connected to a CHI 660E electrochemical workstation (CH Instrument, Chenhua Ltd., Shanghai, China), with the pristine and ECR-3D-TiO_2_ NTAs with an exposed area of 1 cm^2^, Ag/AgCl (3 mol L^−1^ KCl-filled), and Pt mesh as the working, reference, and counter electrode, respectively. The supporting electrolyte was 1 M NaOH (pH = 13.6). The irradiation source was a 500 W Xe lamp (Solar 500, NBet Group Corp., Beijing, China) with calibrated intensity of 100 mWcm^–2^. Moreover, a water filter was used between the lamp and electrochemical cell to remove solution heating from infrared light. An Ocean Optics oxygen sensor system equipped with a FOXY probe (NeoFox Phase Measurement System, Ocean optics, Orlando, FL, USA) was applied to determine the amount of evolved O_2_. The experiment was carried out together with the stability tests. Before the O_2_ measurement, the headspace of the anodic compartment was purged with high purity N_2_ (99.9995%) for 1 h under vigorous stirring. PEC water splitting with O_2_ sensing continued for 180 min at 0.22 V vs. Ag/AgCl, and the O_2_ yield was quantified to calculate the Faradic efficiency. Electrochemical impedance spectroscopy was carried out to understand the charge transfer process between photoelectrodes/electrolyte interfaces. All the measurements were performed under the open circuit condition with the frequency ranging from 0.01 Hz to 100 kHz. Mott-Schottky plots were derived from impedance potential tests conducted at a frequency of 1 kHz in dark conditions.

## 3. Results

### 3.1. Morphological Characterization of the Pristine and ECR-3D-TiO_2_ NTAs

**Figure 1:**
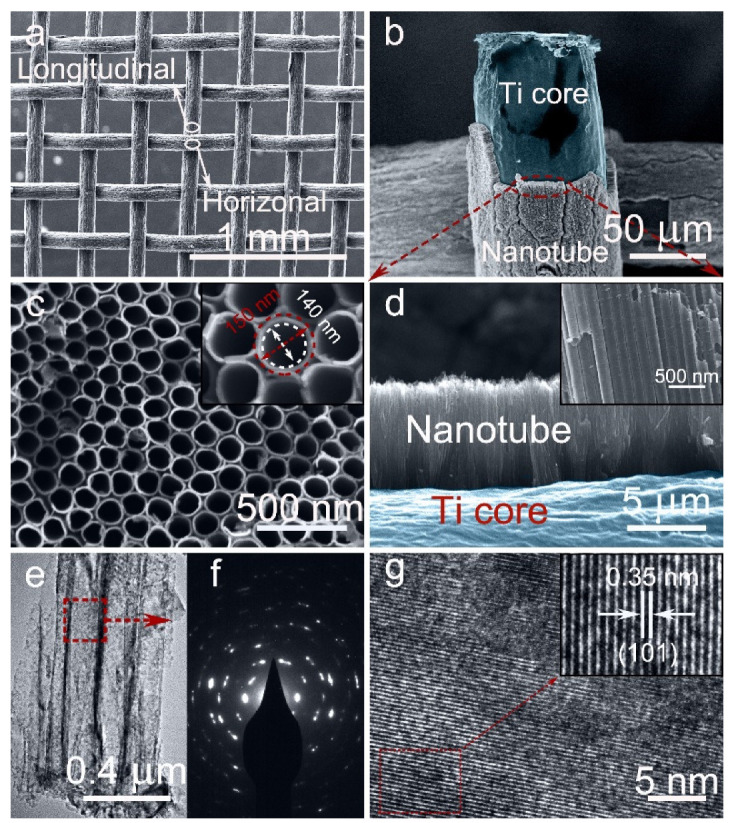
(**a**,**b**) Low-magnification FE-SEM images of the ECR-3D-TiO_2_ NTAs−1.3 V. (**c**,**d**) Corresponding top and cross-sectional view FE-SEM images; Insets: magnified FE-SEM images. (**e**) Low-magnification FE-TEM of the ECR-3D-TiO_2_ NTAs−1.3 V. (**f**,**g**) Selected area electron diffraction (SAED) pattern and HR-TEM image of the area highlighted by the red dashed box in (**e**). Insets: Inverse fast Fourier transform filtered TEM image recorded from the area bounded by the red dashed box in (**g**).

The morphologies of the 3D-TiO_2_ NTAs before and after electrochemical reduction were investigated by FE-SEM. The low-magnification overall FE-SEM image of the ECR-3D-TiO_2_ NTAs−1.3 V displays that the diameter of a single Ti wires is about 0.12 mm and the percentage of the open area of Ti mesh is calculated to approximately 30%, suggesting the higher utilization efficiency of the Ti source (Figure 1a). Figure 1c,d are the magnified FE-SEM images of the area marked by the red ellipse in Figure 1b, which clearly exhibits that TiO_2_ NTAs are radially grown outward around the Ti wires, leading to the formation of 3D-TiO_2_ NTAs. This highly ordered structure can be described by the 3D representation in Figure S1. The top and cross-sectional view FE-SEM images show such ECR-3D-TiO_2_ NTAs with an average diameter of approximately 150 nm, a wall thickness of about 10 nm, and a similar length of 6 μm (Figure 1c,d and Figure S2), which are identical to those of the pristine 3D-TiO_2_ NTAs.

The effect of the electrochemical reduction on the morphologies and microstructures of 3D-TiO_2_ NTAs were further investigated by FE-TEM. From the low-magnification FE-SEM images, all the products possess a tightly packed tubular nanostructures with a mean external diameter of 150 mm, which is consistent with the FE-SEM results (Figure 1e and Figure S3a,d,g). The selected electron diffraction patterns display very similar diffraction patterns, which demonstrate the polycrystalline structures of the 3D-TiO_2_ NTAs before and after electrochemical reduction (Figure 1f and Figure S3b,e,h). In addition, the well-resolved lattice spacing of 0.305 nm are observed in all the products (Figure 1g and Figure S3c,f,i), which corresponds to the {101} plane of anatase TiO_2_ [38,39]. The phase transition of the 3D-TiO_2_ NTAs induced by electrochemical reduction were analyzed by XRD. As shown in Figure S4, all the diffraction peaks match well with crystal structure of the anatase TiO_2_ (JCPDS 21-1272) and metal Ti [38,39]. No other phase is detected, suggesting no change in the lattice structures after electrochemical reduction. The above FE-SEM, FE-TEM and XRD results imply that electrochemical reduction does not destroy the morphology, microstructures or phase of the 3D-TiO_2_ NTAs.

### 3.2. Optical Absorption Properties of the Pristine and ECR-3D-TiO_2_ NTAs

**Figure 2:**
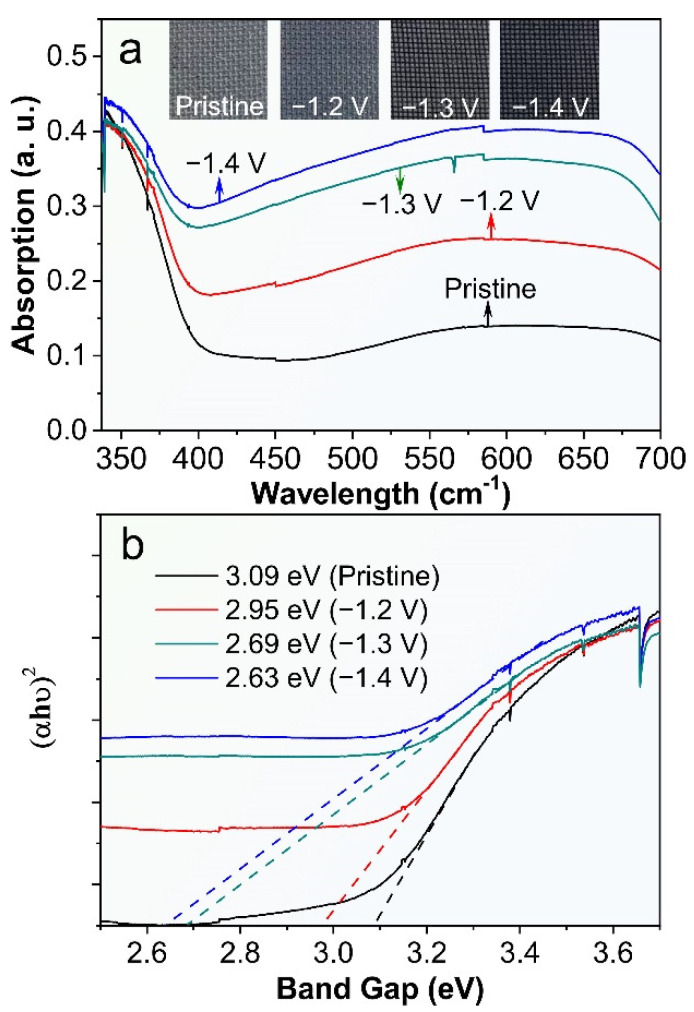
(**a**) UV-vis reflectance spectra and photographs (inset) of the pristine 3D-TiO_2_ NTAs and ECR-3D-TiO_2_ NTAs electrochemically reduced under the different applied bias −1.2, −1.3 and −1.4 V. (**b**) Corresponding curves of the transformed Kubelka–Munk function vs. the energy of light.

We have investigated the UV-vis reflectance spectra of the ECR-3D-TiO_2_ NTAs as a function of external bias applied in the electrochemical reduction and then compared with that of the pristine 3D-TiO_2_ NTAs. Clearly, the pronounced absorption can be clearly observed in the UV region (<390 nm) of all the products, which can be attributed to the intrinsic band-to-band absorption of TiO_2_ [38,39,44]. Compared with the pristine 3D-TiO_2_ NTAs, the visible light absorption (400–800 nm) is significantly enhanced after electrochemical reduction. As the applied bias changes from −1.2 to −1.4 V, the visible light absorption increases gradually, which are further verified by the color variation of the ECR-3D-TiO_2_ NTAs. This implies that the ECR-3D-TiO_2_ NTAs may respond to the visible light region (Figure 2a). Moreover, the bandgaps of the pristine 3D-TiO_2_ NTAs and ECR-3D-TiO_2_ NTAs−1.2, −1.3 and −1.4 V, estimated from the intercept of the tangents to the curves of (αhυ)^2^ vs. photon energy by assuming TiO_2_ as a direct semiconductor, are about 3.09, 2.95, 2.65 and 2.63, respectively (Figure 2b). These results suggest that the electrochemical reduction not only promote the visible light absorption, but also reduce the bandgap of the 3D-TiO_2_ NTAs, which can be ascribed to the presence of the defect state in the bandgap of TiO_2_ created by the O-vacancies. The boosted visible light absorption and bandgap shrinkage means that visible light trapped by the 3D configuration can excite electron-hole pairs and thus effectively improve the PEC water splitting activity of the 3D-TiO_2_ NTAs.

### 3.3. Surface Oxidation State of the Pristine and ECR-3D-TiO_2_ NTAs

**Figure 3:**
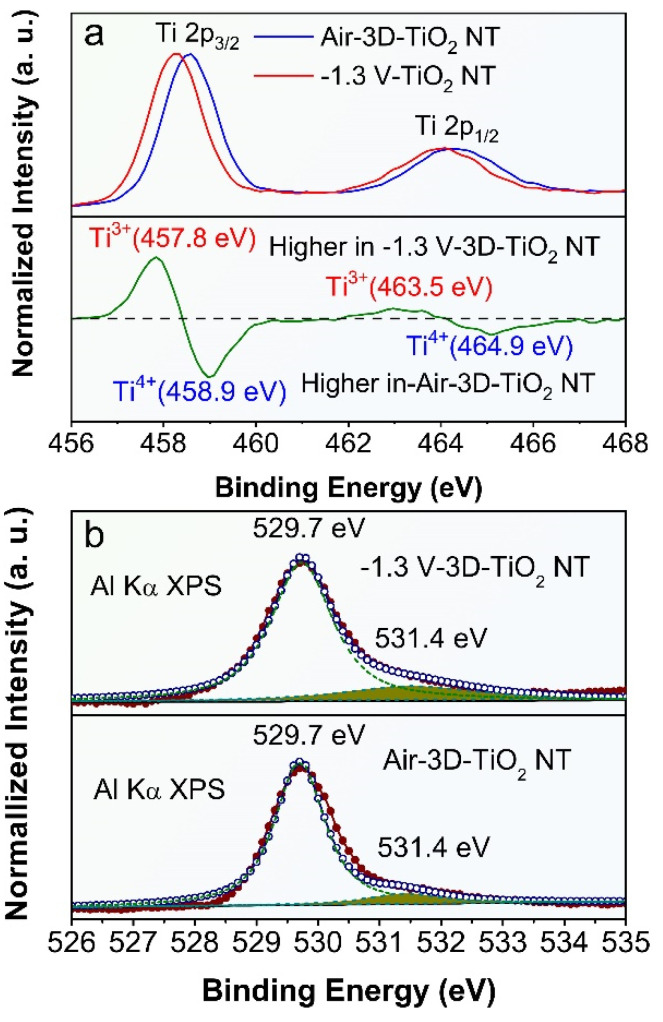
(**a**) Normalized Ti 2p XPS spectra of the pristine 3D-TiO_2_ NTAs and ECR-3D-TiO_2_ NTAs−1.3 V, and their difference spectrum (ECR-3D-TiO_2_ NTAs−1.3 V minus pristine 3D-TiO_2_ NTAs). (**b**) Normalized O1s XPS spectra of the pristine 3D-TiO_2_ NTAs and ECR-3D-TiO_2_ NTAs−1.3 V. The red circles represent the experimental XPS data. The blue circles are the fitting of the experimental data and can be divided into two peaks displayed by the green dashed lines.

To solidify the presence of O-vacancies in the ECR-3D-TiO_2_ NTAs, the chemical composition and surface oxidation states of the pristine 3D-TiO_2_ NTAs and ECR-3D-TiO_2_ NTAs were further examined by XPS. Only Ti, O and C signals are observed in the survey spectra of all the products, which reveals that electrochemical reduction does not introduce other impurities (Figure S5a). For the pristine 3D-TiO_2_ NTAs, the Ti 2p core level spectrum has two peaks centered at 458.3 and 464.1 eV, which are typical for the Ti 2p_3/2_ and 2p_1/2_ peaks of Ti^4+^ in TiO_2_ (Figure 3a) [39,43,50]. After the electrochemical reduction, the Ti 2p_3/2_ and 2p_1/2_ peaks shift to the low binding energy of 457.9 and 463.7 eV, illustrating the different bonding environment of the Ti atom. By subtracting the normalized Ti 2p spectra of the ECR-3D-TiO_2_ NTAs−1.3 V with that of the pristine 3D-TiO_2_ NTAs, two extra peaks at 457.7 and 463.3 eV were observed, which were indexed to the Ti 2p_3/2_ and 2p_1/2_ peaks of Ti^3+^ [39,43,50]. This indicates that O-vacancies are introduced in the ECR-3D-TiO_2_ NTAs−1.3 V. In addition, the O1s spectra of the ECR-3D-TiO_2_ NTAs−1.3 V was also different from that of that of the pristine 3D-TiO_2_ NTAs. In the O1s spectra, the main peak located 529.7 eV is the characteristic peak reported for lattice oxygen of TiO_2_, while other peaks centered at 531.4 eV can be associated with oxygen species absorbed at O-vacancies [39]. As displayed in Figure 3b and Figure S5b, the peaks of area of 531.4 eV of ECR-3D-TiO_2_ NTAs increase gradually with electrochemical reduction bias reducing from −1.2 V to −1.4 V, which suggests that the amount of the O-vacancies increases with the deceasing electrochemical reduction bias. This is why the visible light absorption increases gradually with the electrochemical reduction bias reducing from −1.2 to −1.4 V.

### 3.4. PEC Water Splitting Activity of the Pristine and ECR-3D-TiO_2_ NTAs

**Figure 4:**
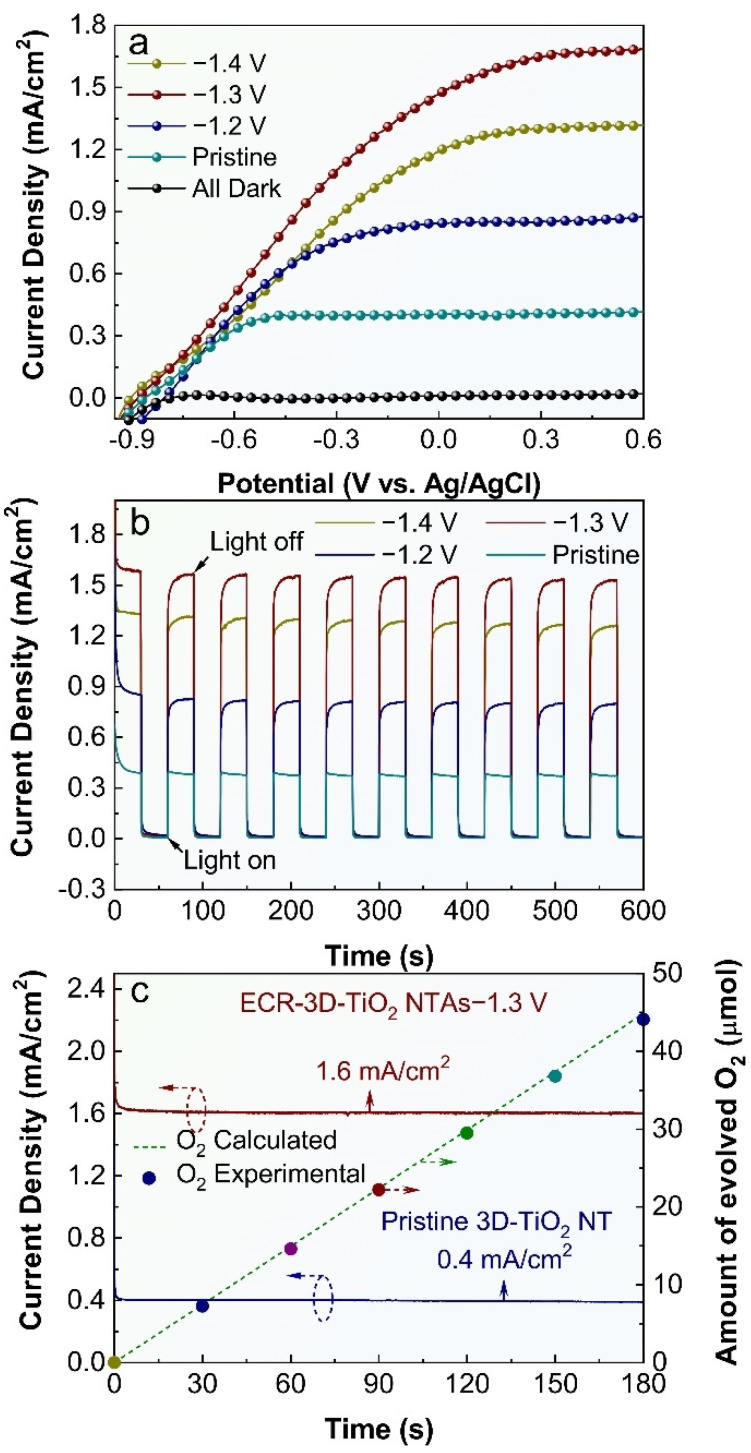
(**a**) Current density vs. voltage (J-V) plots of the pristine 3D-TiO_2_ NTAs and ECR-3D-TiO_2_ NTAs electrochemically reduced under the different applied bias −1.2, −1.3 and −1.4 V. (**b**) Corresponding transient photocurrent responses measured at 0.22 V vs. Ag/AgCl. (**c**) Photocurrent vs. time (*J-t*) curves of the pristine and ECR-3D-TiO_2_ NTAs−1.3 V obtained at 0.22 V vs. Ag/AgCl. The dashed line and colorful circles are the amount of the evolved O_2_ calculated theoretically and detected experimentally of the ECR-3D-TiO_2_ NTAs−1.3 V, respectively.

The influence of the electrochemical reduction bias on the PEC water splitting activity of 3D-TiO_2_ NTAs were also studied, and the results are shown in Figure 4. All the 3D-TiO_2_ NTAs-based photoelectrodes display negligible dark currents in comparison with their respective photocurrents, suggesting no occurrence of the electrocatalytic water splitting. Under irradiation, the photocurrent densities of the ECR-3D-TiO_2_ NTAs increase steeply and are distinctly larger than that of the pristine 3D-TiO_2_ NTAs in the whole potential window from −0.9 to 0.6 V vs. Ag/AgCl, which reveals that the electrochemical reduction can significantly promote the PEC performance of the 3D-TiO_2_ NTAs. Figure 4b compares the transient photocurrent responses of the pristine and ECR-3D-TiO_2_ NTAs measured at 0.22 V vs. Ag/AgCl. It can be seen that all the 3D-TiO_2_ NTAs-based photoelectrodes show excellent sensitivity to the light irradiation. There is a steep rise in current density from almost zero in dark conditions to a stable value upon illumination. In addition, the ECR-3D-TiO_2_ NTAs−1.3 V generate a maximal photocurrent density of 1.6 mA/cm^2^, which is about four times larger than that of the pristine 3D-TiO_2_ NTAs (0.4 mA/cm^2^). This photocurrent density value is superior or comparable to the previously reported values on self-doping TiO_2_ NTAs formed on Ti foil (Table S1) [39,43,46,47,48,51]. This means that the optimal electrochemical reduction bias is −1.3 V, which can be attributed to the two-faced effect of the O-vacancies on the PEC water splitting performance, and will be discussed thoroughly in the following text.

The structural and chemical stability is a critical parameter for a photoelectrode during the PEC water splitting. To assess this property, the photocurrent density vs. time (*J-t*) curves of the pristine and ECR-3D-TiO_2_ NTAs−1.3 V are obtained at 0.22 V vs. Ag/AgCl under continuous illumination (Figure 4c). No sign of decrease in photocurrent densities for the pristine and ECR-3D-TiO_2_ NTAs-1.3 V are detected during the entirely measured 180 min. To further identify whether the observed photocurrents derive from the water splitting reaction, the amount of oxygen evolved from the ECR-3D-TiO_2_ NTAs−1.3 V was determined by a fluorescence sensor. The amount of evolved oxygen increases linearly with test time with unity Faradic efficiency. Figure S6 presents the FE-SEM image and XRD pattern of the ECR-3D-TiO_2_ NTAs−1.3 V after continuous PEC water splitting for 180 min, which prove that the surface morphology and crystal phase of the ECR-3D-TiO_2_ NTAs-1.3 V remains intact. These results sufficiently confirm that excellent stability of the ECR-3D-TiO_2_ NTAs−1.3 V, which is suitable for the potential long-term PEC water splitting application.

**Figure 5:**
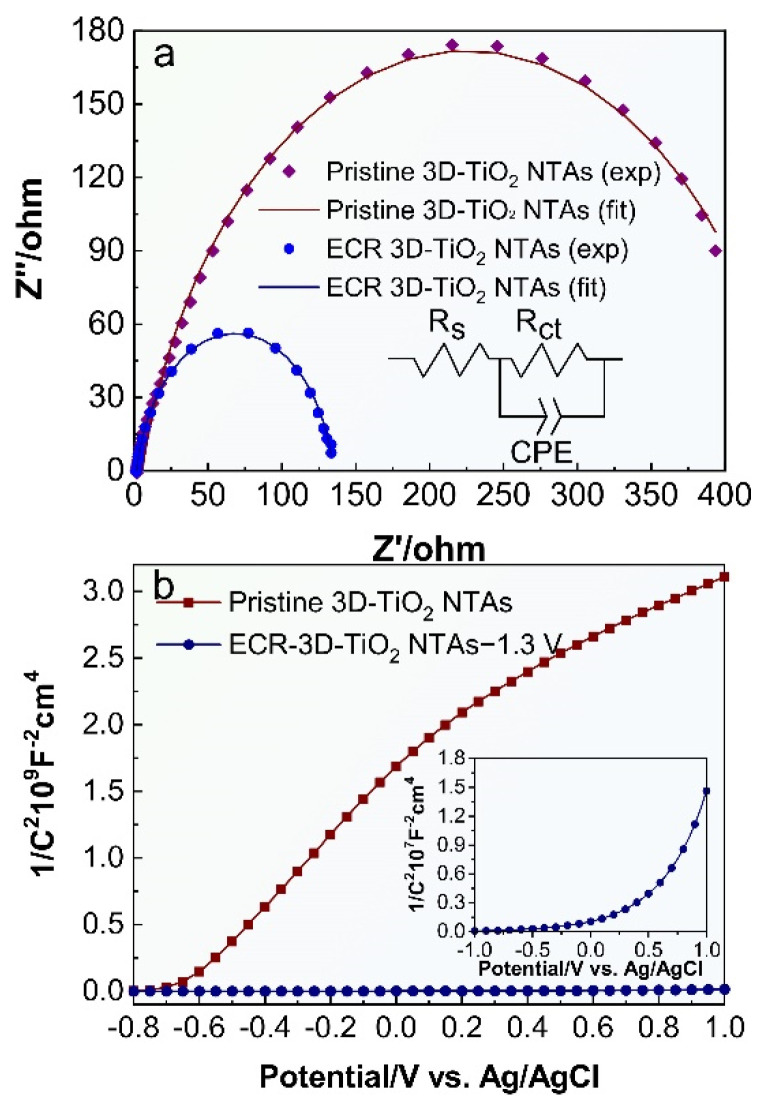
(**a**) Electrochemical impedance spectra of the pristine 3D-TiO_2_ NTAs and ECR- 3D-TiO_2_ NTAs−1.3 V under illumination, and (**b**) Mott–Schottky curves of the pristine 3D-TiO_2_ NTAs and ECR- 3D-TiO_2_ NTAs−1.3 V tested at a frequency of 1 kHz in dark conditions.

To investigate the effect of the electrochemical reduction on the electronic characteristics of 3D-TiO_2_ NTAs, electrochemical impedance spectra (EIS) measurements were performed and the Nyquist plots are shown in Figure 5a, where the scatter points are the original experimental data, and the solid lines are the fitted curves utilizing the equivalent circuit mode in the inset of Figure 5a. It can be clearly seen that the equivalent circuit model fits well with the two samples. In this equivalent circuit model, R_s_ corresponds to the overall series resistance of the circuit, and R_ct_ represents the charge transfer resistance [47,52]. As depicted in Figure 5a, the ECR-3D-TiO_2_ NTAs−1.3 V has a smaller semicircle diameter than the pristine 3D-TiO_2_ NTAs under illumination, suggesting the smaller charge transfer resistance of the ECR-3D-TiO_2_ NTAs−1.3 V. The charge transfer resistance can be obtained by fitting the Nyquist plots with the equivalent circuit model. As expected, the charge transfer resistance R_ct_ of the ECR-3D-TiO_2_ NTAs−1.3 V is reduced from 440.45 to 133.08 Ω, which indicates a more effective separation of the photogenerated electron and hole and/or a faster interfacial charge transfer of the ECR-3D-TiO_2_ NTAs−1.3 V. Moreover, the electrochemical active surface areas of the pristine 3D-TiO_2_ NTAs and ECR-3D-TiO_2_ NTAs−1.3 V are estimated from the capacitive region of cyclic voltammograms (CV). The data shown in Figure S7 reveal that the electrochemically active area of the ECR-3D-TiO_2_ NTAs−1.3 V is only 1.05 times than that of the pristine -3D-TiO_2_ NTAs, indicating that both samples have comparable electrochemically active areas. In addition, the slope of the Mott–Schottky plot collected from the ECR-3D-TiO_2_ NTAs−1.3 V is much smaller than that of the pristine 3D-TiO_2_ NTAs, which suggest an improvement of donor densities (Figure 5b). The donor densities were estimated from the slopes of Mott–Schottky plots using the following equation:

(1) $$N_{D}=-(\frac{2}{e_{0}\varepsilon \varepsilon_{0}}){\left[\frac{d(1/C^{2})}{d(U_{s})}\right]}^{-1}$$

where $e_{0}=-1.6\times 10^{-19}$, $\varepsilon_{0}=8.86\times 10^{-12}$ and ε=48 for the anatase TiO_2_. The calculated donor densities of the pristine and ECR-3D-TiO_2_ NTAs−1.3 V are about 1.03 × 10^19^ and 1.46 × 10^21^ cm^−3^, respectively. The increased the donor density can be attributed to the generation of the O-vacancies that works as electron donors. The increased donor density can effectively boost the transport property of the photogenerated charge carrier, which are of benefit to enhance the PEC water splitting activity. Moreover, the increased donor densities can also shift the Fermi level of the TiO_2_ toward the conduction band, which facilitate the charge separation at the semiconductor–electrolyte interface.

## 4. Discussion

Based on the above experimental results, the boosted photoelectrochemical water splitting performance of ECR-3D-TiO_2_ NTAs can be ascribed to the introduction of the O-vacancies. Firstly, PEC water splitting performance of the photoelectrode largely depend on its capability of effectively absorbing visible light. In the present case, the presence of O-vacancies results in the generation of a new defect energy level near the conduction band, which lead to the bandgap shrinkage, hence being favorable for the visible light harvesting. More importantly, the incident, reflected and/or refracted visible light captured by the 3D configuration is also absorbed by defect energy level near CB created by oxygen vacancy. Secondly, the introduction of the O-vacancies (Ti^3+^) in ECR-3D-TiO_2_ NTAs generally work as electron donors, which leads to the enhanced electronic conductivity and upward shift of the Fermi energy level, thereby facilitating the transfer and separation of photogenerated charge carrier at the semiconductor–electrolyte interface. Nevertheless, the excess O-vacancies may be the recombination centers for photogenerated carriers, hence limiting the generation of photocurrent [53,54]. Therefore, the optimized amount of the O-vacancies is essential to the PEC water splitting performance. The XPS result illustrates that the amount of the O-vacancies increases with deceasing electrochemical reduction bias (Figure 3 and Figure S5). Consequently, it can be included that the ECR-3D-TiO_2_ NTAs−1.4 V may possess excess amount of the O-vacancies (Ti^3+^), which lead to the recombination of photogenerated carriers before reaching the TiO_2_/electrolyte interface. Accordingly, the optimal electrochemical reduction bias is −1.3 V from the perspective of PEC water splitting activity.

## 5. Conclusions

In conclusion, black 3D-TiO_2_ NTAs have been successfully fabricated via an electrochemical reduction and employed as a photoanode for PEC water splitting. The introduction of the O-vacancies results in bandgap shrinkage, which can effectively boost the utilization efficiency of visible light including the incident, reflected and/or refracted visible light captured by the 3D configuration. Moreover, the O-vacancies (Ti^3+^) can work as electron donors, which leads to the enhanced electronic conductivity and upward shift of the Fermi energy level, thereby facilitating the transfer and separation of photogenerated charge carrier at the semiconductor–electrolyte interface. Benefiting from the oxygen vacancy, the optimized photocurrent density of ECR-3D-TiO_2_ NTAs under white light illumination generated the photocurrent density of 1.6 mA/cm^2^ at 0.22 V vs. Ag/AgCl, which is superior or comparable to the previously reported values on self-doping TiO_2_ NTAs formed on Ti foil.

## Data Availability

Data are available from the authors on request.

## Supplementary Materials

The following supporting information can be downloaded at: https://www.mdpi.com/article/10.3390/nano12091447/s1, Figure S1: 3D schematic diagram of the 3D-TiO_2_ NTAs, which clearly exhibits the growth of TiO_2_ NTAs on Ti mesh in a radially outward direction.; Figure S2: FE-SEM image of the pristine 3D-TiO_2_ NTAs, ECR-3D-TiO_2_ NTAs−1.2 V and ECR-3D-TiO_2_ NTAs−1.4 V; Figure S3: FE-TEM image of the pristine 3D-TiO_2_ NTAs, ECR-3D-TiO_2_ NTAs−1.2 V and ECR-3D-TiO_2_ NTAs-1.4 V; Figure S4: XRD patterns of the pristine 3D-TiO_2_ NTAs and ECR-3D-TiO_2_ NTAs electrochemically reduced under different applied bias −1.2, −1.3 and −1.4 V; Figure S5: (a) Survey spectrum of the pristine and ECR-3D-TiO_2_ NTAs electrochemically reduced under the different applied bias −1.2, −1.3 and −1.4 V. (b) O1s XPS spectra of pristine and ECR-3D-TiO_2_ NTAs electrochemically reduced under the different applied bias −1.2, −1.3 and −1.4 V; Figure S6: (a) FE-SEM image and (b) XRD pattern of the ECR-3D-TiO_2_ NTAs−1.3 V after undergoing the PEC water splitting reaction for 180 min. The results obviously show that the morphology of the ECR-3D-TiO_2_ NTAs−1.3 V maintained intact and without observed structural degradation. Figure S7: Cyclic voltammetry (CV) for (a) ECR-3D-TiO_2_ NTAs-1.3 V (b) Pristine-3D-TiO_2_ NTAs under different scan rates. (c) Relative electrochemical surface areas of the ECR-3D-TiO_2_ NTAs−1.3 V and Pristine-3D-TiO_2_ NTAs photoanodes: linear relationship between the capacitive current and scan rate. Table S1: Comparison of the PEC performance for the self-doping TiO_2_ NTAs on formed on Ti foil [39,43,46,47,48,51].

## References

[1] Fu H.C., Varadhan P., Lin C.H., He J.H. (2020). Spontaneous solar water splitting with decoupling of light absorption and electrocatalysis using silicon back-buried junction. Nat. Commun..

[2] Huang D.W., Li L.T., Wang K., Li Y., Feng K., Jiang F. (2021). Wittichenite semiconductor of Cu_3_BiS_3_ films for efficient hydrogen evolution from solar driven photoelectrochemical water splitting. Nat. Commun..

[3] Nandal V., Pihosh Y., Higashi T., Minegishi T., Yamada T., Seki K., Sugiyama M., Domen K. (2021). Probing fundamental losses in nanostructured Ta_3_N_5_ photoanodes: Design principles for efficient water oxidation. Energy Environ. Sci..

[4] Lu Y., Yang Y.L., Fan X.Y., Li Y.Q., Zhou D.H., Cai B., Wang L.Y., Fan K., Zhang K. (2022). Boosting charge transport in BiVO_4_ photoanode for solar water oxidation. Adv. Mater..

[5] Nellist M.R., Laskowski F.A.L., Qiu J.J., Hajibabaei H., Sivula K., Hamann T.W., Boettcher S.W. (2020). Potential-sensing electrochemical atomic force microscopy for in operando analysis of water splitting catalysts and interfaces. Nat. Mater..

[6] Li Y., Mei Q., Liu Z.J., Hu X.S., Zhou Z.H., Huang J.W., Bai B., Liu H., Ding F., Wang Q.Z. (2022). Fluorine-doped iron oxyhydroxide cocatalyst: Promotion on the WO_3_ photoanode conducted photoelectrochemical water splitting. Appl. Catal. B Environ..

[7] Narangari P.R., Narangari R., Butson J.D., Tan H.H., Jagadish C., Karuturi S. (2021). Surface-tailored InP nanowires via self-assembled Au nanodots for efficient and stable photoelectrochemical hydrogen evolution. Nano Lett..

[8] Zhang B.B., Huang X.J., Zhang Y., Lu G.X., Chou L.J., Bi Y.P. (2020). Unveiling the activity and stability origin of BiVO_4_ photoanodes with FeNi oxyhydroxides for oxygen evolution. Angew. Chem. Int. Ed..

[9] Ye S., Shi W.W., Liu Y., Li D.F., Yin H., Chi H.B., Luo Y.L., Ta N., Fan F.T., Wang X.L. (2021). Unassisted photoelectrochemical cell with multimediator modulation for solar water splitting exceeding 4% solar-to-hydrogen efficiency. J. Am. Chem. Soc..

[10] Yang Y., Niu S.W., Han D.D., Liu T.Y., Wang G.M., Li Y. (2017). Progress in developing metal oxide nanomaterials for photoelectrochemical water splitting. Adv. Energy Mater..

[11] Wang W.R., Guo B.D., Dai H.T., Zhao C., Xie G.C., Ma R.P., Akram M.Z., Shan H.Y., Cai C.Z., Fang Z.Y. (2019). Improving the water oxidation efficiency with a light-induced electric field in nanograting photoanodes. Nano Lett..

[12] Wei T.C., Zhu Y.N., Gu Z.N., An X.Q., Liu L.M., Wu Y.X., Liu H.J., Tang J.W., Qu J.H. (2018). Multi-electric field modulation for photocatalytic oxygen evolution: Enhanced charge separation by coupling O-vacancies with faceted heterostructures. Nano Energy.

[13] Samuel E., Joshi B., Kim M.W., Swihart M.T., Yoon S.S. (2020). Morphology engineering of photoelectrodes for efficient photoelectrochemical water splitting. Nano Energy.

[14] Zhang X.M., Zhai P.L., Zhang Y.X., Wu Y.Z., Wang C., Ran L., Gao J.F., Li Z.W., Zhang B., Fan Z.Z. (2021). Engineering single-atomic NiN_4_O sites on semiconductor photoanodes for high-performance photoelectrochemical water splitting. J. Am. Chem. Soc..

[15] Qiu Y.C., Liu W., Chen W., Chen W., Zhou G.M., Hsu P.C., Zhang R.F., Liang Z., Fan S.S., Zhang Y.G. (2016). Efficient solar-driven water splitting by nanocone BiVO_4_-perovskite tandem cells. Sci. Adv..

[16] Yang Q., Du J.Y., Nie X.Q., Yang D.M., Bian L., Yang L., Dong F.Q., He H.C., Zhou Y., Yang H.M. (2021). Magnetic field-assisted photoelectrochemical water splitting: The photoelectrodes gave weaker nonradiative recombination of carrier. ACS Catal..

[17] Hu Y.X., Pan Y.Y., Wang Z.L., Lin T.G., Gao Y.Y., Luo B., Hu H., Fan F.T., Liu G., Wang L.Z. (2020). Lattice distortion induced internal electric field in TiO_2_ photoelectrode for efficient charge separation and transfer. Nat. Commun..

[18] Liu Z.Y., Zhang Q.Q., Zhao T.Y., Zhai J., Jiang L. (2011). 3D vertical arrays of TiO_2_ nanotubes on Ti meshes: Efficient photoanodes for water photoelectrolysis. J. Mater. Chem..

[19] Kołodziej J.K., Chudecka A., Sulka G.D. (2018). 3D nanoporous titania formed by anodization as a promising photoelectrode material. J. Electroanal. Chem..

[20] Liao J.J., Lin S.W., Zhang L., Pan N.Q., Cao X.K., Li J.B. (2012). Photocatalytic degradation of methyl orange using a TiO_2_/Ti mesh electrode with 3D nanotube arrays. ACS Appl. Mater. Interfaces.

[21] Bao R.Y., Zhao Y., Ma F.F., Wu J.H., Xia J.X., Li H. (2022). 3D-TNAs composite electrodes with enhanced visible-light photoelectrocatalytic performance and stability. J. Phys. Chem. Solids.

[22] Liu Z.Y., Wang Q.Y., Cao D.D., Wang Y.J., Jin R.C., Gao S.M. (2020). Vertical grown BiOI nanosheets on TiO_2_ NTs/Ti meshes toward enhanced photocatalytic performances. J. Alloys Compd..

[23] Liu Z.Y., Song Y.D., Wang Q.Y., Jia Y., Tan X.Y., Du X.X., Gao S.M. (2019). Solvothermal fabrication and construction of highly photoelectrocatalytic TiO_2_ NTs/Bi_2_MoO_6_ heterojunction based on titanium mesh. J. Colloid. Interf. Sci..

[24] Li T.T., Wang Z.H., Liu C.C., Tang C.M., Wang X.K., Ding G.S., Ding Y.C., Yang L.X. (2018). TiO_2_ nanotubes/Ag/MoS_2_ meshy photoelectrode with excellent photoelectrocatalytic degradation activity for tetracycline hydrochloride. Nanomaterials.

[25] Jia Y., Liu P.B., Wang Q.Y., Wu Y., Cao D.D., Qiao Q.A. (2021). Construction of Bi_2_S_3_-BiOBr nanosheets on TiO_2_ NTA as the effective photocatalysts: Pollutant removal, photoelectric conversion and hydrogen generation. J. Colloid Interf. Sci..

[26] Bao R.Y., Chen C., Xia J.X., Chen H.Y., Li H. (2019). Controlled synthesis and enhanced photoelectrocatalytic activity of a 3D-TiO_2_ nanotube array/TiO_2_ nanoparticle heterojunction using a combined dielectrophoresis/sol-gel method. J. Mater. Chem. C.

[27] Yang X.C., Chen C. (2016). Cu_2_O sensitized flexible 3D-TiO_2_ nanotube arrays for enhancing visible photo-electrochemical performance. RSC Adv..

[28] Smith Y.R., Subramanian V. (2011). Heterostructural composites of TiO_2_ mesh-TiO_2_ nanoparticles photosensitized with CdS: A new flexible photoanode for solar cells. J. Phys. Chem. C.

[29] Kar A., Smith Y.R., Subramanian V. (2009). Improved photocatalytic degradation of textile dye using titanium dioxide nanotubes formed over titanium wires. Environ. Sci. Technol..

[30] Foo C., Li Y.Y., Lebedev K., Chen T.Y., Day S., Tang C., Tsang S.C.E. (2021). Characterisation of oxygen defects and nitrogen impurities in TiO_2_ photocatalysts using variable-temperature X-ray powder diffraction. Nat. Commun..

[31] Gao J.Q., Xue J.B., Jia S.F., Shen Q.Q., Zhang X.C., Jia H.S., Liu X.G., Li Q., Wu Y.C. (2021). Self-doping surface oxygen vacancy-induced lattice strains for enhancing visible light-driven photocatalytic H_2_ evolution over black TiO_2_. ACS Appl. Mater. Interfaces.

[32] Cheng X., Dong G.J., Zhang Y.J., Feng C.C., Bi Y.P. (2020). Dual-bonding interactions between MnO_2_ cocatalyst and TiO_2_ photoanodes for efficient solar water splitting. Appl. Catal. B Environ..

[33] Paidi V.K., Lee B.H., Ahn D., Kim K.J., Kim Y., Hyeon T., Hyeon T., Lee K.S. (2021). Oxygen-vacancy-driven orbital reconstruction at the surface of TiO_2_ core-shell Nanostructures. Nano Lett..

[34] Meng M., Qin W., Li C.Y., Xu K., Xu L.Y., Li J., Ma L., Liu K.L., Li J.T., Qin N. (2020). Synergistic effect of photonic crystals and oxygen vacancies on photoelectrochemical water splitting of TiO_2_ nanotube. J. Nanoelectron. Optoelectron..

[35] Liu Q.H., He J.F., Yao T., Sun Z.H., Cheng W.R., He S., Xie Y., Peng Y.H., Cheng H., Sun Y.F. (2014). Aligned Fe_2_TiO_5_-containing nanotube arrays with low onset potential for visible-light water oxidation. Nat. Commun..

[36] Liu X.Y., Zhu G.L., Wang X., Yuan X.T., Lin T.Q., Huang F.Q. (2016). Progress in black titania: A new material for advanced photocatalysis. Adv. Energy Mater..

[37] Wei N., Liu Y., Feng M., Lia Z.X., Chen S.G., Zheng Y.B., Wang D.A. (2019). Controllable TiO_2_ core-shell phase heterojunction for efficient photoelectrochemical water splitting under solar light. Appl. Catal. B Environ..

[38] Cui H.L., Zhao W., Yang C.Y., Yin H., Lin T.Q., Shan Y.F., Xie Y., Gua H., Huang F.Q. (2014). Black TiO_2_ nanotube arrays for high-efficiency photoelectrochemical water-splitting. J. Mater. Chem. A.

[39] Meng M., Zhou S.H., Yang L., Gan Z.X., Liu K.L., Tian F.S., Zhu Y., Li C.Y., Liu W.F., Yuan H.L. (2018). Hydrogenated TiO_2_ nanotube photonic crystals for enhanced photoelectrochemical water splitting. Nanotechnology.

[40] Li Z.H., Zhou C., Hua J.H., Hong X.F., Sun C.L., Li H.W., Xu X., Mai L.Q. (2020). Engineering O-vacancies in a polysulfde-blocking layer with enhanced catalytic ability. Adv. Mater..

[41] Lei F.C., Sun Y.F., Liu K.T., Gao S., Liang L., Pan B.C., Xie Y. (2014). O-vacancies confined in ultrathin indium oxide porous sheets for promoted visible-light water splitting. J. Am. Chem. Soc..

[42] Lin T.Q., Yang C.Y., Wang Z., Yin H., Lu X.J., Huang F.Q., Lin J.H., Xie X.M., Jiang M.H. (2014). Effective nonmetal incorporation in black titania with enhanced solar energy utilization. Energy Environ. Sci..

[43] Kang Q., Cao J.Y., Zhang Y.J., Liu L.Q., Xu H., Ye J.H. (2013). Reduced TiO_2_ nanotube arrays for photoelectrochemical water splitting. J. Mater. Chem. A.

[44] Wang G.M., Yang Y., Ling Y.C., Wang H.Y., Lu X.H., Pu Y.C., Zhang J.Z., Tong Y.X., Li Y. (2016). An electrochemical method to enhance the performance of metal oxides for photoelectrochemical water oxidation. J. Mater. Chem..

[45] Chang X., Thind S.S., Chen A.C. (2014). Electrocatalytic enhancement of salicylic acid oxidation at electrochemically reduced TiO_2_ nanotubes. ACS Catal..

[46] Zhang Z.H., Hedhili M.N., Zhu H.B., Wang P. (2013). Electrochemical reduction induced self-doping of Ti3+ for efficient water splitting performance on TiO_2_ based photoelectrodes. Phys. Chem. Chem. Phys..

[47] Song J.N., Zheng M.J., Yuan X.L., Li Q., Wang F.Z., Ma L.G., You Y.X., Liu S.H., Liu P.J., Jiang D.K. (2017). Electrochemically induced Ti^3+^ self-doping of TiO_2_ nanotube arrays for improved photoelectrochemical water splitting. J. Mater. Sci..

[48] Xu C., Song Y., Lu L.F., Cheng C.W., Liu D.F., Fang X.H., Chen X.Y., Zhu X.F., Li D.D. (2013). Electrochemically hydrogenated TiO_2_ nanotubes with improved photoelectrochemical water splitting performance. Nanoscale Res. Lett..

[49] Close T., Tulsyan G., Diaz C.A., Weinstein S.J., Richter C. (2015). Reversible oxygen scavenging at room temperature using electrochemically reduced titanium oxide nanotubes. Nat. Nanotechnol..

[50] Lu X.H., Wang G.M., Zhai T., Yu M.H., Gan J.Y., Tong Y.X., Li Y. (2012). Hydrogenated TiO_2_ nanotube arrays for supercapacitors. Nano Lett..

[51] Li Z.Z., Xin Y.M., Wu W.L., Fu B.H., Zhang Z.H. (2016). Phosphorus cation doping: A new strategy for boosting photoelec trochemical performance on TiO_2_ nanotube photonic crystals. ACS Appl. Mater. Interfaces.

[52] Cheng X., Zhang Y.J., Bi Y. (2019). Spatial dual-electric fields for highly enhanced the solar water splitting of TiO_2_ nanotube arrays. Nano Energy.

[53] Gan J.Y., Lu X.H., Wu J.S., Xie S.L., Zhai T., Yu M.H., Zhang Z.S., Mao Y.C., Wang S.C., Shen Y. (2013). O-vacancies promoting photoelectrochemical performance of In_2_O_3_ nanocubes. Sci. Rep..

[54] Meng M., Yang L., Wu X.L., Gan Z.X., Pan W.Y., Liu K.L., Li C.Y., Qin N., Li J. (2020). Boosted photoelectrochemical performance of In_2_O_3_ nanowires via modulating O-vacancies on crystal facets. J. Alloys Compd..

