# Supplementary material for: Black 3D-TiO2 Nanotube Arrays on Ti Meshes for Boosted Photoelectrochemical Water Splitting
Source: Nanomaterials (Basel). 2022 Apr 24;12(9):1447. doi: 10.3390/nano12091447 (PMC9104132; doi:10.3390/nano12091447)
Supplement: Supplementary file 1 [file nanomaterials-12-01447-s001.zip › nanomaterials-1637293-supplementary.pdf]

nanomaterials

MDPI

Supporting Materials

Black 3D-TiO_2 Nanotube Arrays on Ti Meshes for Boosted Photoelectrochemical Water Splitting

Ming Meng [1,*], Yamin Feng [1], Chunyang Li [1], Zhixing Gan [2,*], Honglei Yuan [1] and Honghui Zhang [1]

[1] School of Physics and Telecommunication Engineering, Zhoukou Normal University, Zhoukou 466001, China; yadan205@126.com (Y.F.); lichunyang98@163.com (C.L.); yuanhenu@163.com (H.Y.); zhanghonghui4714@163.com (H.Z.)

[2] Key Laboratory of Optoelectronic Technology of Jiangsu Province, School of Physical Science and Technology, Nanjing Normal University, Nanjing 210023, China

* Correspondence: mengmingfly@163.com (M.M.); zxgan@njnu.edu.cn (Z.G.)

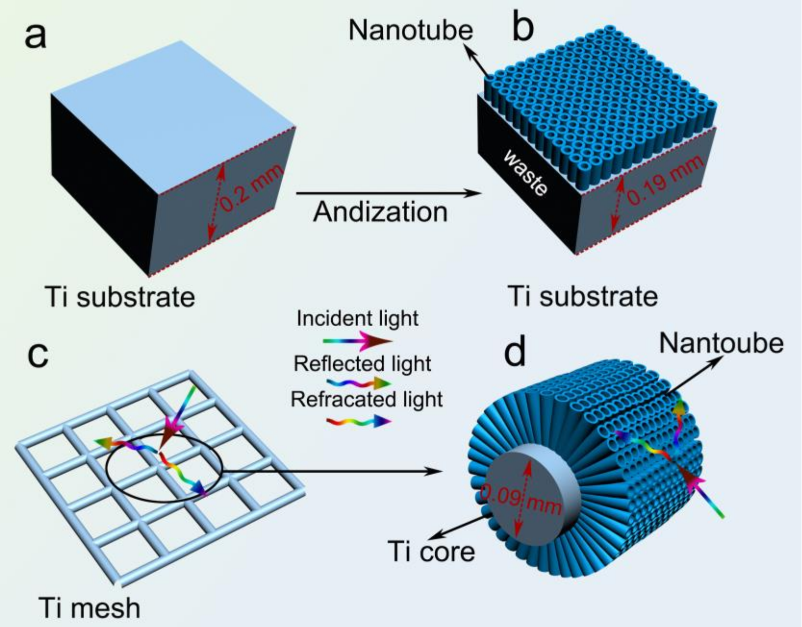

Figure S1. (a) Schematic diagram of the Ti substrate before the electrochemical anodization. (b) The 2D TiO_2 NTAs on Ti substrate. (c) Schematic diagram of the Ti mesh before the electrochemical anodization. (d) 3D schematic diagram of the 3D TiO_2 NTAs, which clearly exhibits the growth of TiO_2 NTAs on Ti mesh in a radially outward direction.

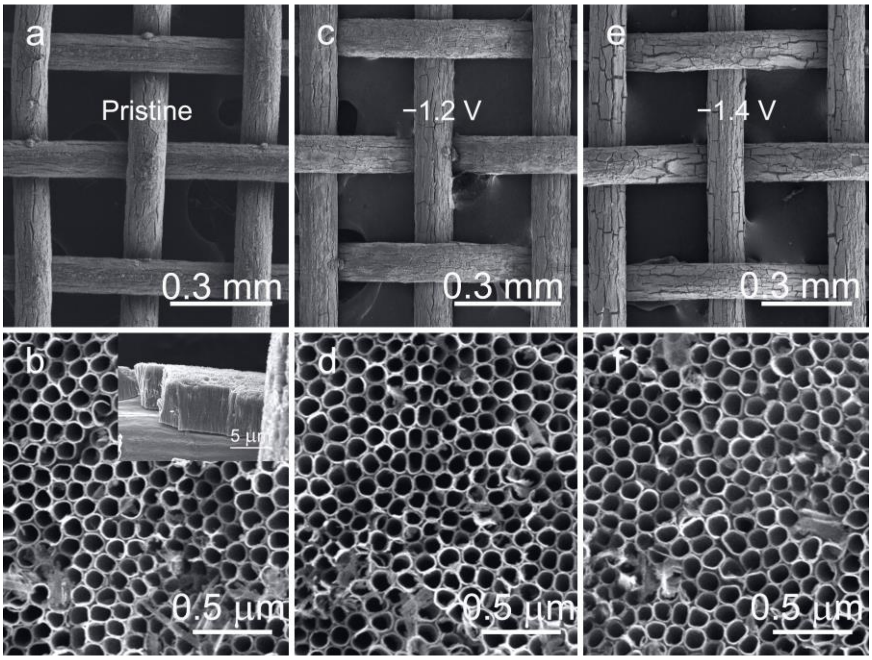

Figure S2. (a, b) FE-SEM image of the pristine 3D-TiO_2 NTAs, Inset of b: cross-sectional view FE-SEM images. (c,d) FE-SEM image of the ECR-3D-TiO_2 NTAs−1.2 V. (e, f) FE-SEM image of ECR-3D-TiO_2 NTAs−1.4 V.

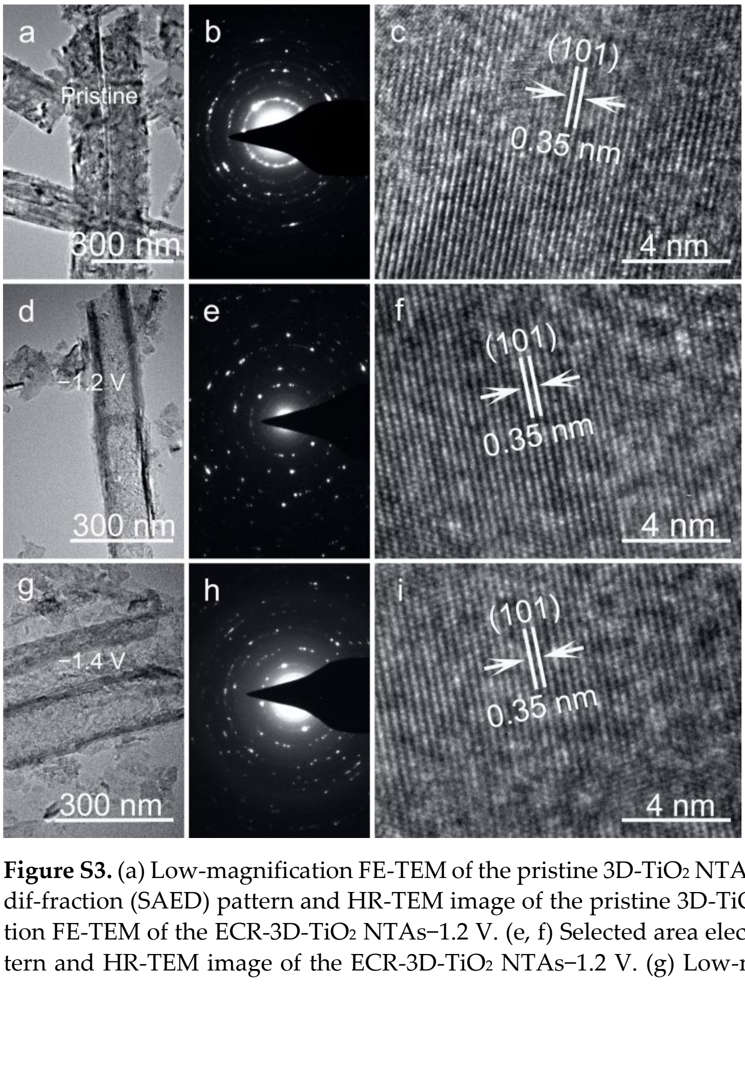

Figure S3. (a) Low-magnification FE-TEM of the pristine 3D-TiO_2 NTAs. (b, c) Selected area electron dif-fraction (SAED) pattern and HR-TEM image of the pristine 3D-TiO_2 NTAs. (d) Low-magnification FE-TEM of the ECR-3D-TiO_2 NTAs−1.2 V. (e, f) Selected area electron dif-fraction (SAED) pattern and HR-TEM image of the ECR-3D-TiO_2 NTAs−1.2 V. (g) Low-magnification FE-TEM of the

ECR-3D-TiO_2 NTAs−1.4 V. (h, i) Selected area electron dif-fraction (SAED) pattern and HR-TEM image of the ECR-3D-TiO_2 NTAs−1.4 V.

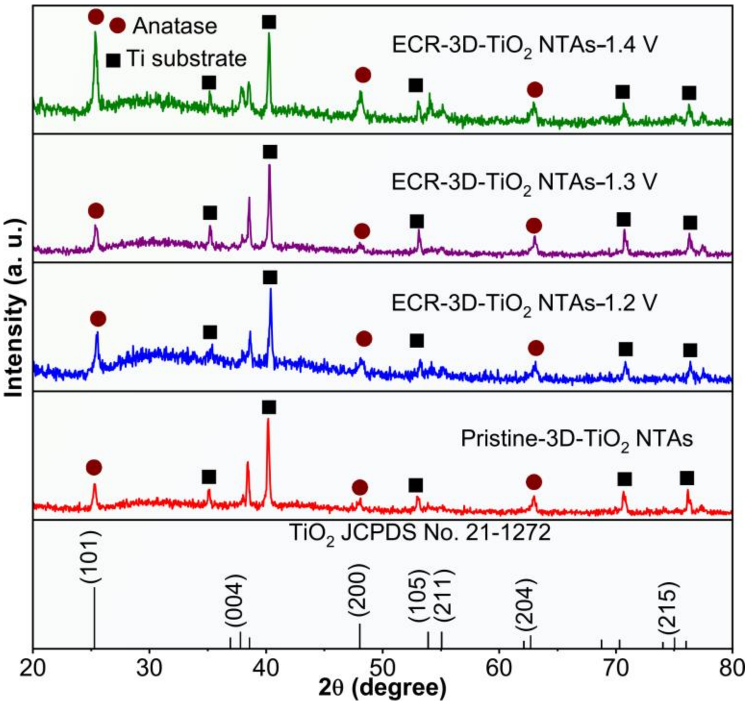

Figure S4. XRD patterns of the pristine 3D TiO_2 NTAs and ECR-3D TiO_2 NTAs electrochemically reduced under different applied bias −1.2, −1.3 and −1.4 V.

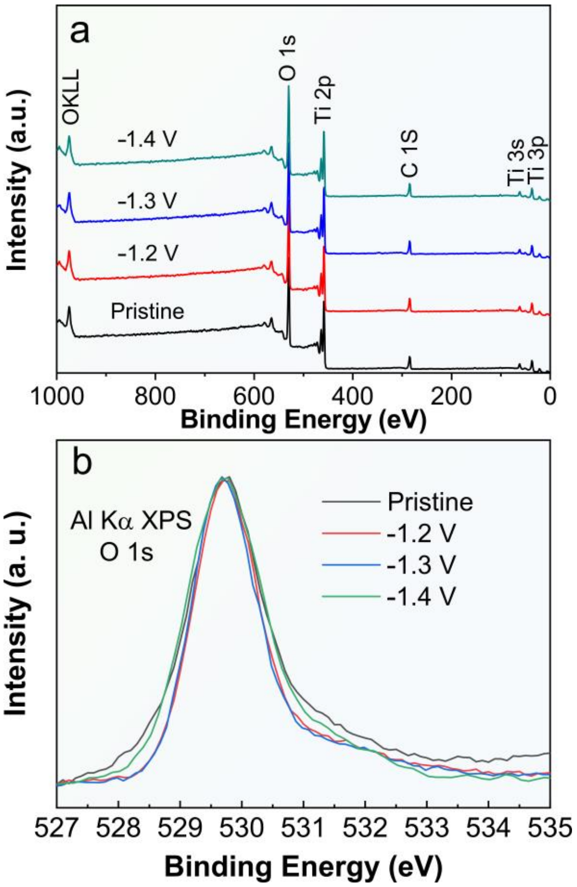

Figure S5. (**a**) Survey spectrum of the pristine and ECR-3D TiO_2 NTAs electrochemically reduced under the different applied bias −1.2, −1.3 and −1.4 V. (**b**) O1s XPS spectra of pristine and ECR-3D TiO_2 NTAs electrochemically reduced under the different applied bias −1.2, −1.3 and −1.4 V.

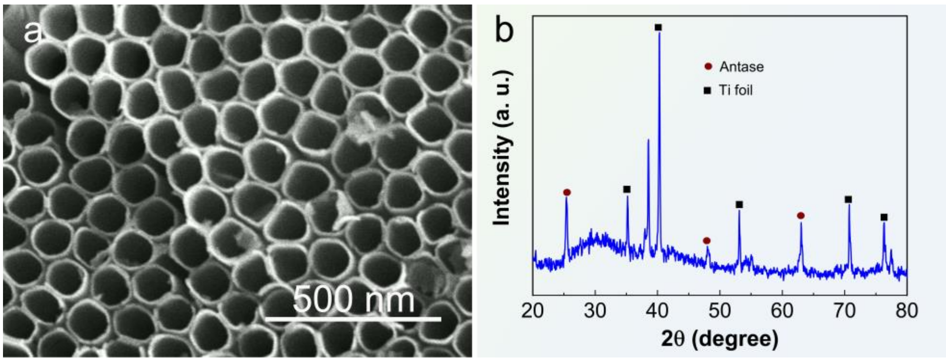

Figure S6. (**a**) FE-SEM image and (**b**) XRD pattern of the ECR-3D TiO_2 NTAs−1.3 V after undergoing the PEC water splitting reaction for 180 min. The results obviously show that the morphology of the ECR-3D TiO_2 NTAs−1.3 V maintained intact and without observed structural degradation.

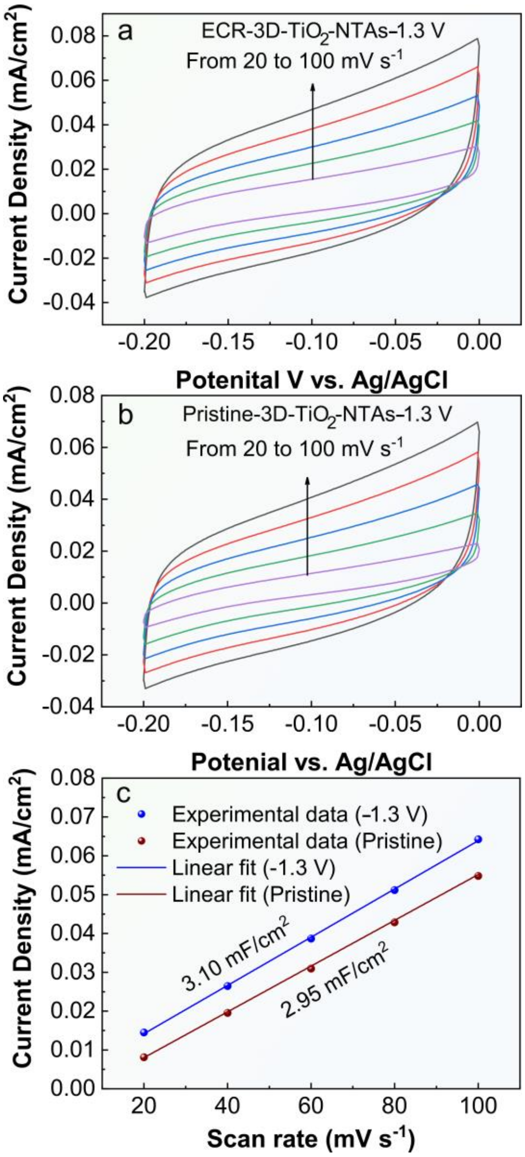

Figure S7. Cyclic voltammetry (CV) for (**a**) ECR-3D TiO_2 NTAs−1.3 V (**b**) Pristine-3D TiO_2 NTAs under different scan rates. (**c**) Relative electrochemical surface areas of the ECR-3D TiO_2 NTAs−1.3

V and Pristine-3D TiO_2 NTAs photoanodes: linear relationship between the capacitive current and scan rate.

Table S1. Comparison of the PEC performance for the self-doping TiO_2 NTAs on formed on Ti foil.

Photoanodes	Photocurrent density At 1.23 V(mA/cm^2)	Enhanced factors	References
Ti^{3+}-self-doped TiO_2 NTAs (2D)	0.52	3.1	[47]
P cation-doped TiO_2 NTAs (2D)	0.78	2.4	[51]
Ti^{3+}-self-doped TiO_2 NTAs (2D)	0.65	2.3	[48]
Reduced TiO_2 NTAs (2D)	0.73	3.0	[43]
Hydrogenated TiO_2 NTAs (2D)	2.1	2.0	[39]
Ti^{3+} self-doped TiO_2 NTAs (2D)	2.8	1.9	[46]
ECR-3D TiO_2 NTAs (3D)	1.6	4.0	This work

References

39. Meng, M.; Zhou, S. H.; Yang, L.; Gan, Z. X.; Liu, K. L.; Tian, F. S.; Zhu, Y.; Li, C. Y.; Liu, W. F.; Yuan, H. L.; Zhang, Y. Hydrogenated TiO_2 nanotube photonic crystals for enhanced photoelectrochemical water splitting. Nanotechnology. **2018**, *29*, 155401.
43. Kang, Q.; Cao, J. Y.; Zhang, Y. J.; Liu, L. Q.; Xu, H.; Ye, J. H. Reduced TiO_2 nanotube arrays for photoelectrochemical water splitting, J. Mater. Chem. A **2013**, 1, 5766-5774.
46. Zhang, Z. H.; Hedhili, M. N.; Zhu, H. B.; Wang, P. Electrochemical reduction induced self-doping of Ti^{3+} for efficient water splitting performance on TiO_2 based photoelectrodes, Phys. Chem. Chem. Phys. **2013**, 15, 15637-15644.
47. Song, J. N.; Zheng, M. J.; Yuan, X. L.; Li, Q.; Wang, F. Z.; Ma, L. G.; You, Y. X.; Liu, S. H.; Liu, P. J.; Jiang, D. K.; Ma, L.; Shen, W. Z. Electrochemically induced Ti^{3+} self-doping of TiO_2 nanotube arrays for improved photoelectrochemical water splitting, J. Mater. Sci. **2017**, 52, 6976-6986.
48. Xu, C.; Song, Y.; Lu, L. F.; Cheng, C. W.; Liu, D. F.; Fang, X. H.; Chen, X. Y.; Zhu, X. F.; Li, D. D. Electrochemically hydrogenated TiO_2 nanotubes with improved photoelectrochemical water splitting performance, Nanoscale Res. Lett. **2013**, 8, 391.
51. Li, Z. Z.; Xin, Y. M.; Wu, W. L.; Fu, B. H.; Zhang, Z. H. Phosphorus cation doping: a new strategy for boosting photoelec trochemical performance on TiO_2 nanotube photonic crystals, ACS Appl. Mater. Interfaces **2016**, 8, 30972-30979.
